# Supplementary material for: Learning from Adaptations to the COVID-19 Pandemic: How Teleconsultation Supported Cancer Care Pathways at a Comprehensive Cancer Center in Northern Italy
Source: Cancers (Basel). 2023 Apr 26;15(9):2486. doi: 10.3390/cancers15092486 (PMC10177335; doi:10.3390/cancers15092486)
Supplement: Supplementary file 1 [file cancers-15-02486-s001.zip › cancers-2357692-supplementary.pdf]

Supplementary file S1. Methodological rules of MDT functioning.

In 2013, our Institution published a specific document on the functioning of MDT groups approved by the Institutional Directorate. The document was revised twice, id est in 2015 and 2019. The document describes the rules that MDT members adopt to discuss all the cases.

Each MDT group defines a set of criteria used to identify which cases can be discussed, at what stage of CP the case discussion takes place, the frequency of meetings, the location and professionals involved. Professionals involved in the MDT group should use the Institute's IT devices and software to plan the cases to be discussed, explain each case, and report, as well as record, the final MDT recommendation. The clinical case is presented by the referring clinician or otherwise, in his or her absence, by a delegate in possession of all clinical information relevant to the discussion of the case at the MDT meeting. At the end of MDT meeting, the MDT recommendation is registered in an electronic medical record and shared with the patient and all professionals involved in the care pathway the patient will face. Each MDT member is responsible for the recommendation according to his/her filed of expertise.
If the referring clinician of the case is absent at the MDT discussion and disagrees with the decision made, the case should be discussed again. If there is no agreement among MDT members during the discussion, differences of opinion should be tracked and documented so that any further exploration, consultation and discussion can proceed. MDT members may attend the meeting in person at the designated meeting location or via video call.

MDT members are coordinated by a Teamleader whose responsibilities include (i) convening the group and coordinating its meetings; (ii) verifying the presence/absence of MDT members and soliciting the presence of absent members; (iii) verifying that documentation related to the discussion of cases is properly prepared and appropriate; and (iv) monitoring and evaluating the functioning of the MDT group by referring to the Directorate's CP Manager in case critical issues. The overall MDT functioning is periodically monitored by specific indicators.
